# Rice bran extract supplement improves sleep efficiency and sleep onset in adults with sleep disturbance: A randomized, double-blind, placebo-controlled, polysomnographic study

**DOI:** 10.1038/s41598-019-48743-8

**Published:** 2019-08-26

**Authors:** Min Young Um, Hyejin Yang, Jin Kyu Han, Jin Young Kim, Seung Wan Kang, Minseok Yoon, Sangoh Kwon, Suengmok Cho

**Affiliations:** 1 Research division of functional food functionality, Korea Food Research Institute, Wanju, 55365 Republic of Korea; 2 Seoul Sleep Center, Seoul, 06041 Republic of Korea; 3 Department of Nursing, Seoul National University, Seoul, 03080 Republic of Korea; 4 S&D Research and Development Institute, Cheongju, 28156 Republic of Korea; 5 Department of Food Science and Technology, Pukyong National University, Busan, 48513 Republic of Korea

**Keywords:** Nutrition, Randomized controlled trials

## Abstract

We previously reported that rice bran extract supplement (RBS) administration to mice decreased sleep latency and induced non-rapid eye movement (NREM) sleep via inhibition of the histamine H_1_ receptor. Based on this, we performed the first clinical trial to investigate whether RBS would be beneficial to subjects with disturbed sleep. We performed a randomized, double‐blinded, placebo‐controlled, 2-week study. Fifty subjects with sleep disturbance were enrolled and received either RBS (1,000 mg/day) or placebo. Polysomnography was performed, and Pittsburgh Sleep Quality Index, Epworth Sleepiness Scale (ESS), and Fatigue Severity Scale were administered at the initiation and termination of the study. Compared with the placebo, RBS led to significant polysomnographic changes, including decreased sleep latency (adjusted, P = 0.047), increased total sleep time (P = 0.019), and improved sleep efficiency (P = 0.010). Additionally, the amount of stage 2 sleep significantly increased in the RBS group. When adjusted for caffeine intake, wakefulness after sleep onset, total wake time, and delta activity tended to decrease in the RBS group. RBS administration decreased ESS scores. There were no reported serious adverse events in both groups. RBS improved sleep in adults with sleep disturbance. Trial registration: WHO ICTRP, KCT0001893.

## Introduction

Insomnia is the most common sleep-related complaint, and approximately 10–40% of adults experience insomnia at some point during their lifetime^1–3^. In addition, 5–10% of the adult population has chronic insomnia^4^. The number of patients diagnosed with insomnia in South Korea has been increasing over the years. According to an epidemiologic study, the prevalence of insomnia in South Korea was 25.3% in women and 20.2% in men, with the highest prevalence among the older population (>65 years)^5^. The major classes of drugs for the treatment of insomnia include γ-Aminobutyric acid type A (GABA_A_)-benzodiazepine (BZD) receptor agonists, melatonin receptor agonists, antidepressants, and antihistamines^6^. Recently, the orexin receptor antagonist was approved by the FDA for the treatment of insomnia^7^. However, efforts for identifying the ideal sedative-hypnotics that will provide better results without adverse effects are still ongoing. Meanwhile, natural sleep aids or herbal are becoming more general for the treatment of insomnia due to their safety and efficacy^8^. Various medicinal plants, including valerian, hop, jasmine, kava, lemon balm, passion flower, and lavender, have been commercially used as hypnotic ingredients in dietary supplements for insomnia^9^.

In recent studies, an epidemiology study in Japan showed a rice consumption was associated with sleep quality^10^. Byun *et al*.^11^, reported that the supplementation of fermented rice germ extract containing GABA for 4 weeks increased the Pittsburgh Sleep Quality Index (PSQI) total score compared with the baseline, and decreased the sleep latency. We recently reported that similar to doxepin used in the treatment of insomnia, rice bran extract supplement (RBS) potentiated pentobarbital-induced sleep in mice^12^. RBS counteracted caffeine-induced sleep disturbance in mice^12^. In addition, we demonstrated that the RBS promotes sleep via inhibition of histamine H_1_ receptors (H_1_R)^12,13^. The RBS decreased sleep latency and increased non-rapid eye movement (NREM) sleep in wildtype mice; whereas, in histamine H_1_R knockout mice, it did not produce sleep-promoting effects. Moreover, r-oryzanol, β‐sitosterol, campesterol, stigmasterol, ferulic acid, and (±)‐α‐tocopherol, which are rice bran constituents, increased the sleeping time in ICR mice with pentobarbital-induced sleep^14^. These results suggest that the RBS may be a promising agent for the improving sleep although this results are mainly from animal model.

Based on the above rationale and potential for the RBS to improve sleep, we aimed to investigate for the first time in a systematic manner whether sleep among subjects with disturbed sleep can be improved through RBS administration. To this end, we designed the first RBS randomized, double-blind, placebo-controlled trial employing overnight polysomnography (PSG) recordings as well as sleep-related standardized scales.

## Results

### Subjects and compliance

**Figure 1:**
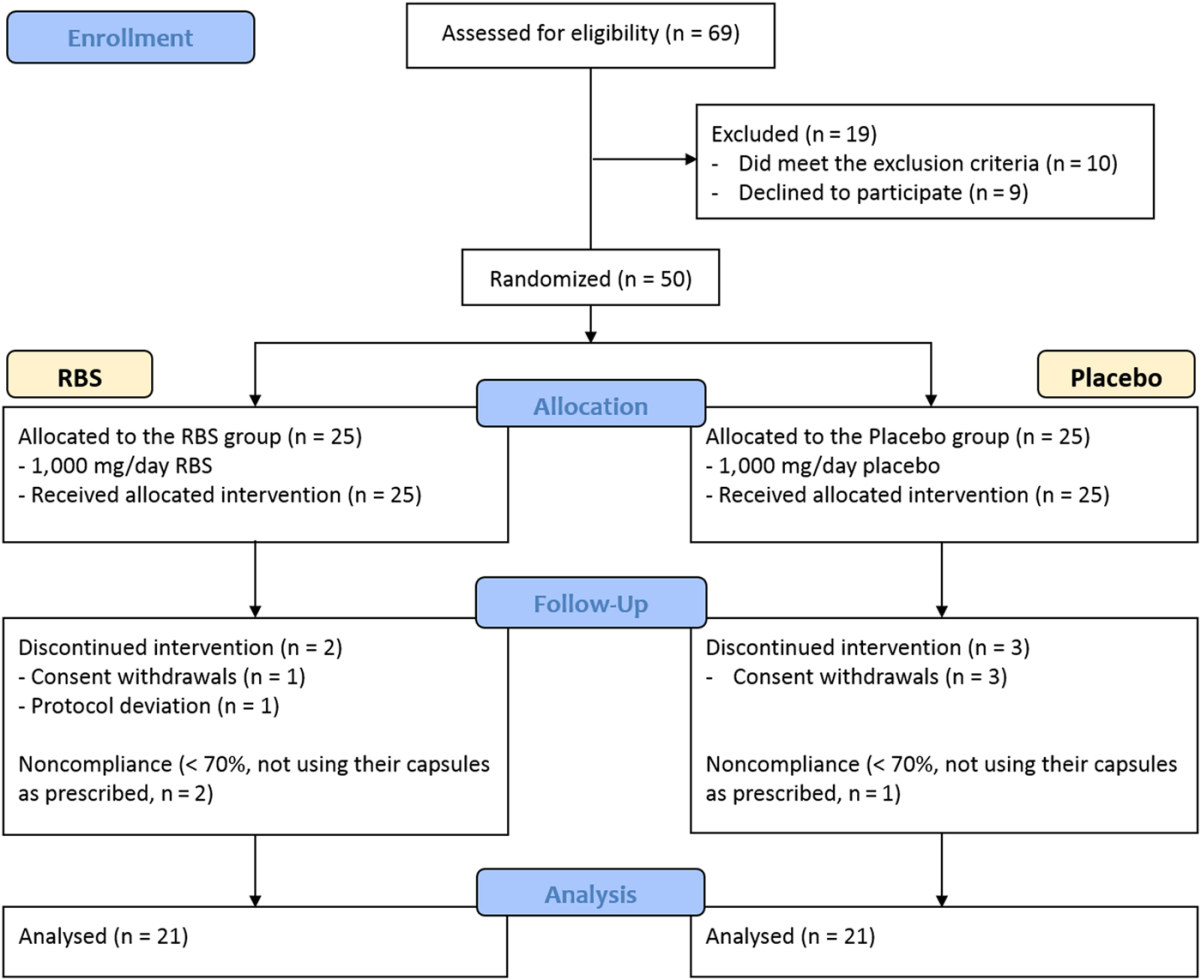
CONSORT flow chart-trial of study protocol.

As described in Fig. 1, 69 participants were enrolled in the present clinical trial, 19 participants were excluded based on the exclusion criteria (n = 10) and because they declined participation (n = 9). A total of 50 participants were randomized, of these, 45 participants completed the study. There were three dropouts in the placebo group and two dropouts in the RBS group. Three subjects were excluded from analysis due to noncompliance with the study procedure.

The baseline characteristics of the participants are shown in Table 1. At baseline, there were no significant differences between the placebo and RBS groups in terms of age, sex, body mass Index (BMI), and blood pressure. The average caffeine intake at the initial visit (week 0), as calculated from self-reports, was significantly different between the groups (P = 0.045). And, the intake levels of total energy and macronutrients were not different between the two groups (Supplementary Table S1). Compliance to study product intake was comparable between the groups: 97.5% in the placebo group and 94.6% in the RBS group (P = 0.35).

**Table 1:** Baseline characteristics of the study participants.

	Placebo (n = 25)	RBS (n = 25)	P value^a^
Age (years)	46.0 ± 14.0	41.8 ± 13.5	0.272
Sex (male/female)	7/18	11/14	0.239
Weight (kg)	62.2 ± 10.3	64.0 ± 11.5	0.560
BMI (kg/m^2^)	23.2 ± 3.5	23.0 ± 3.0	0.790
BMI < 25 kg/m^2^, (n, %)	16 (64)	18 (72)	0.544
BMI ≥ 25 kg/m^2^, (n, %)	9 (36)	7 (28)	
SBP (mmHg)	123.2 ± 19.7	118.0 ± 15.0	0.302
DBP (mmHg)	80.1 ± 10.2	76.4 ± 9.7	0.197
Caffeine intake (mg/day)	78.3 ± 53.9	128.5 ± 107.6	0.045

Data are expressed as the mean ± standard deviation or number.

^a^Independent t-tests or Chi-squared tests were used to determine significant differences between the groups.

DBP: diastolic blood pressure; SBP: systolic blood pressure.

### Self-report sleep and fatigue outcomes

The Pittsburgh Sleep Quality Index (PSQI), Epworth Sleepiness Scale (ESS), and Fatigue Severity Scale (FSS) scores were evaluated at baseline and at the endpoint of the study. As shown in Table 2, the PSQI total scores significantly decreased within both groups compared with the baseline scores, but no significant changes were observed between the groups. When compared to the baseline scores, the ESS scores significantly decreased in the RBS group, but this effect was not observed in the placebo group. Therefore, the change in the ESS scores was significantly greater in the RBS group than in the placebo group (P = 0.027). In the FSS evaluation, no differences in fatigue severity were revealed between the two groups.

**Table 2:** PSQI, ESS, and FSS scores in the placebo and RBS groups before and after the 2-week intervention.

Parameter		Placebo (n = 21)	RBS (n = 21)	P value^a^ (Unadjusted)	P value^b^ (Adjusted)
PSQI	At Baseline	7.0 ± 2.1	7.4 ± 2.5	0.545	
	At 2 weeks	4.9 ± 2.1*	5.6 ± 2.7*	0.343	
	Difference	−2.1 ± 1.8	−1.8 ± 2.3	0.657	0.510
ESS	At Baseline	6.7 ± 3.1	10.1 ± 4.4	0.007	
	At 2 weeks	7.0 ± 4.8	7.7 ± 5.1*	0.643	
	Difference	0.3 ± 3.5	−2.3 ± 3.9	0.027	0.045
FSS	At Baseline	3.4 ± 1.4	3.5 ± 1.2	0.804	
	At 2 weeks	3.0 ± 1.4	3.5 ± 1.3	0.287	
	Difference	−0.3 ± 0.9	0.0 ± 0.8	0.198	0.164

Data are expressed as the mean ± standard deviation.

*Within-group comparison, paired t-test, P < 0.05.

^a^P values refer to independent t-tests, compared with the placebo group.

^b^P values are for the analysis of covariance; adjusted for caffeine intake as a covariate.

PSQI scores, with higher scores indicate worse sleep quality.

ESS scores, higher scores indicate greater sleepiness.

FSS scores, higher scores indicate more fatigue severity.

### PSG outcomes

As shown in Table 3, after 2 weeks of intervention, the RBS led to a significant increase in sleep efficiency (SE) and total sleep time (TST) compared with the placebo (placebo group *vs* RBS group, −1.6 ± 5.9% *vs* 2.9 ± 7.7%, P = 0.037; −7.8 ± 21.0 *vs* 11.7 ± 29.9 min, P = 0.019). Additionally, wakefulness after sleep onset (WASO) and total wake time (TWT) decreased by 34.4% and 30.4%, respectively, following RBS administration compared with the placebo, although the difference was not significant (P = 0.071 and P = 0.072, respectively). Baseline levels of caffeine intake were significantly different between the two groups. In addition, numerous studies have reported that caffeine can cause serious sleep problems^15^. Therefore, we controlled the analyses for caffeine intake. When sleep parameters were adjusted for caffeine intake, the SE and TST still showed a significant change between groups (P = 0.019 and P = 0.010, respectively). In particular, by adjusting for caffeine intake, the change in sleep latency (SL) was significantly different between the RBS and placebo groups (P = 0.047).

**Table 3:** Objective sleep parameters analysed by PSG.

Parameter		Placebo (n = 21)	RBS (n = 21)	P value^a^ (Unadjusted)	P value^b^ (Adjusted)
SE, %	At Baseline	92.6 ± 4.8	91.4 ± 8.8	0.562	
	At 2 weeks	91.0 ± 6.2	94.3 ± 4.0	0.051	
	Difference	−1.6 ± 5.9	2.9 ± 7.7	0.037	0.019
TST, min	At Baseline	362.6 ± 16.0	356.4 ± 34.4	0.460	
	At 2 weeks	354.8 ± 24.02	368.1 ± 16.1	0.042	
	Difference	−7.8 ± 21.0	11.7 ± 29.9	0.019	0.010
SL, min	At Baseline	7.8 ± 5.3	9.7 ± 11.3	0.499	
	At 2 weeks	10.7 ± 11.7	6.5 ± 4.6	0.140	
	Difference	2.9 ± 12.9	−3.1 ± 11.7	0.122	0.047
REM sleep latency, min	At Baseline	96.8 ± 43.4	92.4 ± 54.4	0.770	
	At 2 weeks	86.5 ± 45.6	76.9 ± 40.0	0.473	
	Difference	−10.4 ± 58.1	−15.5 ± 56.8	0.774	0.983
WASO, min	At Baseline	20.9 ± 16.5	24.1 ± 25.5	0.635	
	At 2 weeks	24.5 ± 20.7	15.8 ± 14.8	0.126	
	Difference	3.5 ± 19.9	−8.3 ± 23.3	0.084	0.071
TWT, %	At Baseline	5.4 ± 4.2	6.9 ± 6.5	0.638	
	At 2 weeks	6.3 ± 5.3	4.1 ± 3.8	0.126	
	Difference	0.9 ± 5.1	−2.1 ± 6.0	0.084	0.072
Stage 1, %	At Baseline	5.9 ± 2.2	7.4 ± 3.5	0.103	
	At 2 weeks	6.1 ± 3.6	5.7 ± 2.4*	0.721	
	Difference	0.2 ± 4.1	−1.6 ± 3.5	0.125	0.117
Stage 2, %	At Baseline	66.5 ± 5.9	65.3 ± 7.6	0.571	
	At 2 weeks	64.0 ± 7.3	68.4 ± 4.3	0.022	
	Difference	−2.5 ± 7.2	3.1 ± 8.0	0.022	0.020
Stage 3, %	At Baseline	0.5 ± 2.2	0.0 ± 0.0	0.329	
	At 2 weeks	0.6 ± 2.7	0.0 ± 0.0	0.329	
	Difference	0.1 ± 0.5	0.0 ± 0.0	0.329	0.361
REM sleep, %	At Baseline	19.8 ± 5.0	18.7 ± 6.5	0.528	
	At 2 weeks	20.3 ± 5.4	20.1 ± 5.5	0.917	
	Difference	0.5 ± 6.3	1.4 ± 5.9	0.611	0.450
Delta wave, %	At Baseline	29.6 ± 5.0	28.8 ± 4.7	0.611	
	At 2 weeks	29.5 ± 5.9	31.6 ± 9.2	0.387	
	Difference	−0.1 ± 5.5	2.8 ± 10.1	0.262	0.067
RDI	At Baseline	33.6 ± 16.6	29.1 ± 12.2	0.317	
	At 2 weeks	36.5 ± 19.6	29.7 ± 12.4	0.186	
	Difference	2.9 ± 9.5	0.6 ± 5.8	0.357	0.256
AHI	At Baseline	29.7 ± 18.7	22.9 ± 14.9	0.198	
	At 2 weeks	33.0 ± 21.4	23.9 ± 15.8	0.122	
	Difference	3.3 ± 9.9	0.9 ± 6.3	0.366	0.361
Total AI	At Baseline	29.8 ± 13.8	26.6 ± 9.6	0.373	
	At 2 weeks	31.2 ± 15.1	26.7 ± 10.2	0.258	
	Difference	1.4 ± 7.9	0.10 ± 6.5	0.577	0.353

Data are expressed as the mean ± standard deviation.

*Within-group comparison, paired t-test, P < 0.05.

^a^P values refer to independent t-tests, compared with the placebo group.

^b^P values refer to the analysis of covariance, adjusted for caffeine intake as a covariate.

To better understand the effects of RBS on sleep, we additionally analysed the stage of sleep, as well as delta activity in NREM sleep. As expected, compared with the placebo group, RBS administration increased the amount of stage 2 sleep (P = 0.022), but not of stage 1 and stage 3 sleep and rapid eye movement (REM) sleep. When adjusted for caffeine intake, significant changes in stage 2 sleep were still observed (P = 0.020). In addition, delta wave activity showed an enhancing trend in the RBS group (adjusted, P = 0.067).

There was no significant difference regarding the change in Apnea-Hypopnea Index (AHI), respiratory disturbance index (RDI), total arousal index (AI) between the two groups during the experimental period.

### Serum cytokine levels

Table 4 shows the changes in inflammatory cytokines in the RBS and placebo groups. Compared to the placebo, RBS administration had no significant effect on the levels of IL-1β and TNF-α after the 2-week intervention period.

**Table 4:** Concentration of inflammatory cytokines in serum of participants with sleep disturbance administered RBS or placebo for 2 weeks.

Parameter		Placebo (n = 21)	RBS (n = 21)	P value^a^ (Unadjusted)	P value^b^ (Adjusted)
IL-1β (pg/mL)	At Baseline	0.48 ± 1.16	0.45 ± 1.31	0.932	
	At 2 weeks	0.49 ± 0.91	0.38 ± 0.52	0.630	
	Difference	0.03 ± 1.03	−0.06 ± 1.16	0.783	0.672
TNF-α (pg/mL)	At Baseline	1.48 ± 1.78	1.07 ± 0.71	0.327	
	At 2 weeks	1.56 ± 1.76	1.01 ± 0.32	0.166	
	Difference	0.08 ± 2.26	−0.06 ± 0.75	0.785	0.617

Data are expressed as the mean ± standard deviation.

^a^P values refer to independent t-tests, compared with the placebo group.

^b^P values refer to the analysis of covariance, adjusted for caffeine intake as a covariate.

### Safety evaluation

Adverse events (AEs) are listed in Supplementary Table S2. There were no serious AEs during the study. We documented 16 reported AEs (Placebo = 10; RBS = 6), most of which were transient or mild in severity, but the distribution was not significantly different between the groups. All participants had results within normal limits in the laboratory test.

## Discussion

Rice bran contains rich bioactive components such as γ‐oryzanol, tocopherols, tocotrienols, phytosterols, fatty acids, and phenolic compounds. According to a study by Tu *et al*.^14^, ferulic acid potentiated pentobarbital-induced sleep by shortening sleep latency and prolonging sleeping time in a dose-dependent manner. Additionally, Deng *et al*.^16^ reported that ferulic acid has a sedative-hypnotic effect by affecting neurotransmitter levels (serotonin, dopamine, norepinephrine, and GABA) in the cerebrum, brain stem, and the cerebellum of mice. Consistently, in our previous study, rice bran constituents such as β‐sitosterol, campesterol, stigmasterol, (±)‐α‐tocopherol, and ferulic acid affected sleep in a pentobarbital‐induced sleep test compared with doxepin hydrochloride as a positive control^8^. Furthermore, in the results of the animal study using H_1_R knockout mice, oral administration of RBS was shown to have sedative and hypotonic effects in mice by inhibiting H_1_R^13^. Brain histamine is responsible for regulating the sleep-wake cycle^17^. Histaminergic neurons are exclusively located in the posterior hypothalamus and they are reportedly involved in various physiological functions through H_1_, H_2_, H_3_, and H_6_ receptors. Among them, H_1_R has a pivotal role in the regulation of the sleep-wake cycle. Antihistamines, which are H_1_R antagonists, exert significant sleep-promoting and/or wake-promoting effects. In this regard, the sleep-promoting effect of the RBS is completely dependent on H_1_R antagonism.

In the present randomized clinical trial, we assessed the effects of RBS standardized r-oryzanol 4.5 mg/g for adults with disturbed sleep. As mentioned, this study included 50 adults with sleep problems. The number of subjects was similar to that included in other studies that evaluated the sleep efficacy of natural products such as valerian, hop, and ginseng^18–21^. In addition, in systematic review and meta-analysis studies for valerian, trials based on PSG included less than 20 subjects^22,23^. Considering that this human study was based on PSG, the sample size is reasonable.

The RBS administrated to subjects was well tolerated and showed significant improvements in PSG-defined sleep onset, maintenance, and duration in this model of transient insomnia. In our results, there were significant improvements in SL and TST as well as SE. In addition, WASO and TWT tended to decrease with RBS administration. However, a subjective evaluation of sleep quality by a self-report questionnaire (PSQI), was unable to confirm a significant effect in the RBS group. PSG is the most representative method in sleep medicine and is a comprehensive recording of the physiological function changes that occur during the sleep state^24^. An evaluation in a general adult community population by Buysse *et al*.^25^ showed that the PSQI score was also not associated with specific PSG measures. According to a study by Mondal *et al*.^26^, among participants who had abnormal PSQI scores above 5, there were no statistically significant differences observed for most PSG measured parameters. Although our findings revealed that RBS administration did not significantly affect subjective quality of sleep, this is an important finding given the burden associated with sleep onset and maintenance‐associated insomnia. Moreover, these findings are consistent with evidence form another recently published *in vivo* study on RBS^13^. These results suggested that the RBS might improve sleep continuity through shortening of sleep latency and attenuating arousals, leading to an increase in TST.

A sleep stage was also affected by RBS administration. In the present study, the change in stage 2 sleep was increased in the RBS group compared with the placebo group. Further, in the within group comparisons, subjects under RBS administration had decreased stage 1 sleep compared to baseline, whereas no significant changes were observed in the placebo group. Moreover, the PSG results showed that delta activity in the RBS group had an increasing tendency compared with that in the placebo group. Electroencephalograms delta (0.5–4.0 Hz) activity is an indicator of depth or intensity of NREM sleep^27^. Sleeping pills such as BZD agonists actually reduce the amount of delta waves, the essential feature of deep sleep. For example, diazepam produces an increase in the quantity of sleep, but a decrease in sleep quality^28^. Our data indicate that the RBS increased the duration of NREM sleep to a level similar to that observed in physiological sleep, which consequentially showed as an increase in TST without changing the duration of REM sleep. Considering these findings together, it appears reasonable to propose that increasing NREM sleep by RBS administration may be beneficial to individuals with sleep disturbances.

In contrast, daytime impairment is another important component of insomnia. Additionally, sleep disorders and impaired sleep quality are often related to sleepiness and fatigue^29^. Here, we indirectly confirmed the sleep-promoting effect of RBS through measuring daytime sleepiness. As a result, it was found that daytime sleepiness was less experienced by the subjects that received RBS. However, the fatigue score was not affected.

Sleep plays an important role in immune function. Accumulated evidence suggested that sleep may regulate the transcription and translation of inflammatory network^30^. Several studies have also reported increased levels of pro-inflammatory markers such as IL-1β, TNF-α, and IL-6 in the peripheral blood mononuclear cell following sleep deprivation in humans^31,32^. These inflammatory signals lead to the activation of astrocyte and microglia with the subsequent release of pro-inflammatory cytokines and signalling molecules^33^. Nevertheless, our results showed that circulating IL-1β and TNF-α levels did not change by RBS administration. However, in this study, we recruited subjects with disturbed sleep, not patients; therefore, the RBS maybe not have had a beneficial effect on serum cytokines levels.

The present study has some limitations. We observed in overnight PSG study that sleep parameters (SL, SE, TST, and stage 2 sleep) were significantly reduced after RBS administration; however, this was only evaluated across 2 nights in the sleep laboratory. Second, pharmacokinetic analysis is important to elucidate for how RBS affects sleep after intake. Although in the previous animal study, we confirmed the underlying mechanism in that RBS inhibited H_1_R, further study is required to achieve a comprehensive understanding of how the metabolized RBS or its active compounds act in the human brain with impaired sleep. Despite these limitations, this study provided evidence that RBS may improve sleep disturbance.

In conclusion, this is the first study showing that supplementation with RBS may improve sleep onset and maintenance in sleep‐disturbed adults. Therefore, RBS is expected to improve sleep in healthy individuals who experience sleep disturbances.

## Methods

### Participants

The research protocols were approved by the Institutional Review Board of Hanman University (IRB No. 15-03-04-0925; Date 25/09/2015, Daejeon, Republic of Korea). All participants were provided with a full explanation of the study procedure and signed informed consent before entering the clinical trial. All methods were conducted according to relevant guidelines and approved protocol by the Institutional Review Board of Hannam University. This study was registered in the World Health Organization International Clinical Trials Registry Platform (KCT0001893, Date 21/04/2016).

The participants were recruited from the Seoul Sleep Center (Seoul, Korea) over a period of 8 months (November 2015 to June 2016) through advertisements. Subjects with self-reported impaired sleep quality participated in this study. Inclusion criteria were: (1) generally healthy adults ≥ 18 years of age, (2) a score of  ≥ 5 on PSQI, indicating overall disturbed sleep, (3) Subject who voluntarily agrees to participate and signs in the informed consent form^34^. Exclusion criteria were: (1) subjects with sleep disorders or severe insomnia, (2) subjects employed in shift work or having irregular sleeping habits, (3) trans-meridian travel within 4 weeks prior to the first visit, (4) subjects with current major illness (involving the heart, kidneys, liver, cerebrovascular system, gallbladder, or cancer among others), (5) subjects with neurological or psychiatric disorders, (6) individuals addicted to drugs or alcohol, (7) subjects using drugs or dietary supplements that could affect sleep (e.g., sedatives, hypnotics, antidepressants, over-the-counter sleep aids, and herbal products), (8) subjects who consumed functional foods containing rice bran extract, (9) excessive alcohol use (≥ 3 drinks/day or ≥ 6 drinks/week) and caffeine (≥ 3 caffeinated drinks/day), (10) subject who had participated in other human studies within 1 month before the screening visit, (11) subjects with cognitive impairment, (12) pregnant or lactating women, (13) subjects who are deemed ineligible by the investigator to participate in this trial.

### Study design

This study was designed as a 2-week, randomized, double-blind, placebo-controlled, clinical trial. A computer-generated random sequence was used for random allocation and the subjects were randomly allocated to two groups: “RBS” and “Placebo” groups. For the 2 weeks of the intervention, the subjects in the RBS group received 1,000 mg of RBS (3 capsules/day) and the subjects in the Placebo group, a matching placebo (maltodextrin, 3 capsules/day) without food 30–60 min before bedtime. The daily dosage and treatment duration of RBS (1,000 mg) were selected according to the result of the pilot study, clinical trials evaluating the efficacy of natural sleep aids such as valerian, and the previous study in which reported the sleep-promoting effect of RBS in mice^12,13,35–38^. During the intervention study period, the subjects were asked to maintain their usual lifestyle including normal diet and physical activity and to not ingest excessive alcohol, caffeine, over-the-counter (valerian, melatonin and hop, etc.), dietary supplements containing rice bran, and cooked rice bran. To prevent selection bias, the study participants, investigators, and laboratory staff were all blinded to treatment assignment. Participants were educated to bring all remaining supplement at the endpoint of the study, and excluded from further analysis if they consumed less than 70% of the prescribed dose. The investigators asked all participants whether any adverse events had been experienced since the previous visit, and all AEs were documented in the case report form.

### RBS and placebo preparation

The RBS (lot no. SD-RB-002) was provided by S&D Co., Ltd. (Cheongju, Korea) as previously described^13^. Briefly, rice bran (*Oryza sativa* L.) was extracted with ethanol-water solution for 8 hours at 40 °C. Then, the extracts were concentrated under reduced pressure. Finally, after mixing the concentrated solution, d-α-tocopherol, polysorbate 20, sodium caseinate, and dextrin, this mixture was dried and powdered. The RBS was standardized to 4.5 mg/g of γ-oryzanol. Placebo capsules were composed of maltodextrin and a yellow-colour additive. The RBS and placebo capsule were packaged in an indistinguishable manner and labelled with the subject’s number.

### Anthropometric and dietary assessment

Personal characteristics including demographics and disease history were obtained from each subject. The participants’ height was self-reported, and the weight was measured using E-body 205 (Jawan Medical, Kungsan, Korea). The BMI was calculated by dividing the body weight (kg) by squared height (m^2^). Obesity was defined as BMI > 25 kg/m^2^^39^. Blood pressure and pulse were measured with a sphygmomanometer (Omron Matsusaka, Tokyo, Japan). General characteristics of the study subjects were collected through face-to-face interviews.

Dietary intake was estimated by a 3-day food records (2 weekdays and 1 weekend day) at before and after intervention period for both groups. Dietary intake data were analysed using the Computer Aided Nutritional analysis program (CAN‐Pro 4.0, Korea Nutrition Society, Seoul, Korea).

Caffeine intake was calculated on the basis of the analysed results of the amount of caffeine in the main source of caffeine performed by Korea National Institute of Food and Drug Safety Evaluation in 2014^40,41^. If caffeine values were not available on the database, the food product labels were utilized. When it was not found in the database and food label, default values were used. Default values are the assigned caffeine values by food category.

### Subjective questionnaires

The PSQI is a well-validated measure of an individual’s subjective sleep quality. It comprises seven domains, including subjective sleep quality, sleep latency, sleep duration, habitual sleep efficiency, sleep disturbances, the use of sleeping medication, and daytime dysfunction^34^. Daytime sleepiness was measured using the Korean version of the ESS^42^. The FSS score is composed of nine items developed to assess disabling fatigue^43^. Higher average scores indicate greater severity of fatigue symptoms. The questionnaires were completed by subjects at baseline and at the end of the 2-week intervention period.

### PSG

Sleep was objectively assessed by PSG (Embla, Flaga, Reykjavik, Iceland). PSG was performed twice at baseline (Night 1) and at the end of the 2-week intervention period (Night 2) by a trained technician. During the screening period, participants were provided with a sleep diary in which they recorded their sleeping and waking times for 1 week before PSG evaluations. The start time for the PSG recording was scheduled within ± 30 min of the participant’s median bedtime as recorded in the sleep diary. PSG was recorded for 8 hours. The participants were asked to maintain their regular bedtimes and wake times throughout all experimental nights.

Overnight PSG data were collected at the Seoul Sleep Center using Embla (Flaga, Reykjavik, Iceland) and assessed according to standard criteria using 30-s epochs^44^. The following parameters were measured to describe objective changes in sleep architecture: SE, SL, latency to REM sleep, TST, TWT, WASO, total amount of all stages (stage 1–4 NREM sleep and REM sleep), AHI, RDI, and total AI.

### Biochemical analyses

Blood samples were obtained at the baseline and the end of the intervention period. The serum was stored at −80 °C until biochemical analysis. IL-1β and TNF-α levels in serum were analysed using commercial ELISA kits (R&D systems, Abingdon, UK). These assays were then performed according to the manufacturer’s protocols. Safety assessments were based on electrocardiography, haematology, urinalysis, blood biochemistry tests, and pulse rate and blood pressure results at screening, baseline, and the end of the intervention.

### Sample size estimation and statistical analysis

Considering an α = 0.05 and a power of 80%, the sample size was calculated to be 20 per group based on the information for sleep efficiency; anticipating an approximate drop-out rate of 20% during the study course, 25 subjects were recruited in each group. Data are presented as the mean ± SD. A chi-square test was performed to assess group differences at baseline in the frequencies of categorized variables. Within-group comparisons of before and after treatment were performed using paired t-tests. Between-group data were analysed using an independent t-test for group comparisons. Moreover, we used analysis of covariance to compare the mean of post-intervention outcomes between the two groups by adjustment for caffeine intake as a covariate. The safety analysis set included all subjects who received at least one dose of RBS or placebo and had any evaluable safety data. Statistical procedures were performed using the SPSS 20.0 statistical package (SPSS Inc., Chicago, IL, USA). Statistically, significance was considered at P value less than 0.05.

## Supplementary information

Supplementary tables

## Data Availability

The datasets generated during the current study are available from the corresponding author on reasonable request.
