# Supplementary material for: Rice bran extract supplement improves sleep efficiency and sleep onset in adults with sleep disturbance: A randomized, double-blind, placebo-controlled, polysomnographic study
Source: Sci Rep. 2019 Aug 26;9:12339. doi: 10.1038/s41598-019-48743-8 (PMC6710429; doi:10.1038/s41598-019-48743-8)
Supplement: Supplementary file 1 — Supplementary tables [file 41598_2019_48743_MOESM1_ESM.docx]

**Rice bran extract supplement improves sleep efficiency and sleep onset in adults with sleep disturbance: A randomized, double-blind, placebo-controlled, polysomnographic study**

Min Young Um^1^, Hyejin Yang^1^, Jin Kyu Han^2^, Jin Young Kim^3^, Seung Wan Kang^3^, [Minseok Yoon](https://onlinelibrary.wiley.com/action/doSearch?ContribAuthorStored=Yoon%2C+Minseok)^1^, Sangoh Kwon^4^, Suengmok Cho^5,*^

^1^Research division of functional food functionality, Korea Food Research Institute, Wanju 55365, Republic of Korea

^2^Seoul Sleep Center, Seoul 06041, Republic of Korea

^3^Department of Nursing, Seoul National University, Seoul 03080, Republic of Korea

^4^ S&D Research and Development Institute, Cheongju 28156, Republic of Korea

^5^Department of Food Science and Technology, Pukyong National University, Busan 48513, Republic of Korea

* Corresponding author: Suengmok Cho

Associate professor, PhD

Department of Food Science and Technology

Pukyong National University

Busan 48513, Republic of Korea

[scho@pknu.ac.kr](mailto:scho@pknu.ac.kr)

**Supplementary Tale S1.** Daily nutrient intake at baseline and 2 weeks

| **Parameter** | | **Placebo**  **(n = 21)** | **RBS**  **(n = 21)** | **P value^1^** |
| --- | --- | --- | --- | --- |
| Calories, kcal | At Baseline | 1711.1 ± 387.5 | 1743.4 ± 367.6 | 0.784 |
|  | At 2 weeks | 1750.4 ± 398.1 | 1739.9 ± 360.5 | 0.929 |
| Carbohydrates, g | At Baseline | 257.5 ± 70.0 | 252.8 ± 50.2 | 0.804 |
|  | At 2 weeks | 268.2 ± 61.8 | 246.9 ± 61.0 | 0.267 |
| Lipids, g | At Baseline | 48.4 ± 21.4 | 50.4 ± 18.2 | 0.744 |
|  | At 2 weeks | 48.1 ± 18.1 | 51.9 ± 12.9 | 0.445 |
| Protein, g | At Baseline | 65.1 ± 18.4 | 70.9 ± 23.8 | 0.382 |
|  | At 2 weeks | 66.0 ± 20.8 | 74.1 ± 21.7 | 0.222 |
| Total dietary fibre, g | At Baseline | 20.7 ± 6.9 | 19.9 ± 4.2 | 0.634 |
|  | At 2 weeks | 20.9 ± 7.5 | 19.2 ± 6.3 | 0.423 |

Data are expressed as the mean ± standard deviation.

^1^ Independent *t*-tests were used to determine significant differences between the groups.

**Supplementary Tale S2.** Summary of all AEs during intervention

| Any AEs | **Placebo**  **(n=25)**  n (%) | **RBS**  **(n = 25)**  n (%) |
| --- | --- | --- |
| Subjects with ≥ 1 AEs | 9 (36) | 6 (24) |
| Infections | 1 (4) | 0 (0) |
| Headaches | 1 (4) | 1 (4) |
| Dysmenorrhea | 0 (0) | 1 (4) |
| Omodynia | 1 (4) | 0 (0) |
| GI symptoms | 2 (8) | 3 (12) |
| Daytime drowsiness | 1 (4) | 0 (0) |
| Coryza | 1 (4) | 0 (0) |
| Nocturia | 1 (4) | 0 (0) |
| Subconjunctival haemorrhage | 0 (0) | 1 (4) |
| Rash | 1 (4) | 0 (0) |
| Fracture of lower leg | 1 (4) | 0 (0) |
